# Evaluating the Time Toxicity of Cancer Treatment in the CCTG CO.17 Trial

**DOI:** 10.1200/OP.22.00737

**Published:** 2023-03-07

**Authors:** Arjun Gupta, Christopher J. O'Callaghan, Liting Zhu, Derek J. Jonker, Ralph P.W. Wong, Bruce Colwell, Malcolm J. Moore, Christos S. Karapetis, Niall C. Tebbutt, Jeremy D. Shapiro, Dongsheng Tu, Christopher M. Booth

**Affiliations:** ^1^ University of Minnesota, Minneapolis, MN; ^2^ Canadian Cancer Trials Group, Kingston, ON, Canada; ^3^ The Ottawa Hospital Cancer Centre, ON, Canada; ^4^ CancerCare Manitoba, Winnipeg, MB, Canada; ^5^ Dalhousie University, Halifax, NS, Canada; ^6^ Princess Margaret Cancer Centre, Toronto, ON, Canada; ^7^ Flinders University, Adelaide, SA, Australia; ^8^ Austin Health, Melbourne, VIC, Australia; ^9^ Cabrini Health, Malvern, VIC, Australia; ^10^ Department of Oncology, Queen's University, Kingston, ON, Canada

## Abstract

The time spent in pursuing treatments for advanced cancer can be substantial. We have previously proposed a pragmatic and patient-centered metric of these time costs—which we term time toxicity—as any day with physical health care system contact. This includes outpatient visits (eg, bloodwork, scans, etc), emergency department visits, and overnight stays in a health care facility. Herein, we sought to assess time toxicity in a completed randomized controlled trial (RCT).

**METHODS:**

We conducted a secondary analysis of the Canadian Cancer Trials Group CO.17 RCT that evaluated weekly cetuximab infusions versus supportive care alone in 572 patients with advanced colorectal cancer. Initial results reported a 6-week improvement in median overall survival (OS) with cetuximab (6.1 *v* 4.6 months). Subsequent analyses reported that benefit was restricted to patients with *K-ras* wild-type tumors. We calculated patient-level time toxicity by analyzing trial forms. We considered days without health care contact as home days. We compared medians of time measures across arms and stratified results by *K-ras* status.

**RESULTS:**

In the overall population, median time toxic days were higher in the cetuximab arm (28 *v* 10, *P* < .001) although median home days were not statistically different between arms (140 *v* 121, *P* = .09). In patients with *K-ras*–mutated tumors, cetuximab was associated with almost numerically equal home days (114 days *v* 112 days, *P* = .571) and higher time toxicity (23 days *v* 11 days, *P* < .001). In patients with *K-ras* wild-type tumors, cetuximab was associated with more home days (186 *v* 132, *P* < .001).

**CONCLUSION:**

This proof-of-concept feasibility study demonstrates that measures of time toxicity can be extracted through secondary analyses of RCTs. In CO.17, despite an overall OS benefit with cetuximab, home days were statistically similar across arms. Such data can supplement traditional survival end points in RCTs. Further work should refine and validate the measure prospectively.

## INTRODUCTION

Despite progress over the past few decades, most individual treatments for advanced cancer are associated with modest survival benefits (often < 2 months as median).^1^ The amount of time spent in pursuing these treatments can be substantial.^2-4^ Patients spend time in frequent outpatient visits to meet with a clinician, for bloodwork, imaging, infusions and procedures, seeking acute care and follow-up, and rehabilitation care. They also spend time in travel, in waiting rooms, and on logistics such as coordinating care (eg, on the phone with insurance companies, etc) and filling prescriptions.^2,3,5^ Patients' loved ones—friends, family, and community members—spend additional time in accompanying and supporting patients.^6^ Increasingly, oncology as a discipline is recognizing the need to describe where and how patients spend their time, not just how much added time (survival) is associated with pursuing a treatment.^3,5^ We conceptualize the time burdens of pursuing a treatment as the time toxicity of the treatment. This information is pertinent to all patients, and most so to people with advanced cancer, who have to make treatment decisions in the context of limited time. Although it is possible to retrospectively calculate time toxicity associated with a treatment from claims or electronic health record data,^2,7,8^ this approach does not allow accurate comparisons across treatments (because of confounding).

We have previously extracted and compared the time toxicity in clinical trials using trial publications and protocols.^3^ We have demonstrated that the loss of time incurred by pursuing a treatment may be more than the modest survival benefit associated with that treatment.^9,10^ This work is limited by the lack of available patient-level information, forcing us to construct the hypothetical course of an average patient in the trial.^3^

Clinical trials, the gold standard to prospectively assess and compare efficacy, provide a unique opportunity to compare the time toxicity of treatments. Oncology clinical trials already collect vast amounts of data. Cooperative group trials additionally often collect measures of resource utilization.^11^ Innovative data collation approaches may enable patient-level analyses of completed trials. If feasible, this approach would allow researchers to leverage existing trial data, while encouraging future oncology trials to incorporate time toxicity as a prospective end point. We undertook the following proof-of-concept study to determine the feasibility of assessing time toxicity in a completed cooperative group randomized clinical trial (RCT).

## METHODS

### Study Background, Procedures, and Efficacy/Safety Data

We conducted a secondary analysis of the Canadian Cancer Trials Group CO.17 open-label RCT (ClinicalTrials.gov identifier: NCT00079066). CO.17 recruited participants in Canada, Australia, and New Zealand during 2003-2005. Eligible patients had advanced colorectal cancer and the disease had progressed on fluoropyrimidine, irinotecan, and oxaliplatin. Patients were randomly assigned 1:1 to receive weekly cetuximab infusions or supportive care alone. Patients in both arms underwent clinical assessments every 4 weeks, with imaging every 8 weeks. The primary end point was overall survival (OS).

A total of 572 patients were randomly assigned. Initial results published in 2007 reported a 6-week improvement in median OS with cetuximab (6.1 months *v* 4.6 months, hazard ratio for death, 0.77; *P* = .005).^12^ The median duration of cetuximab treatment was 8.1 weeks. Cetuximab was associated with less deterioration in health status. Cetuximab was associated with a higher incidence of rash, infection, confusion, and pain. Follow-up publications in 2008 and 2009 reported the association between *K-ras* mutation status and cetuximab efficacy—patients with a tumor-bearing mutated *K-ras* did not benefit from cetuximab, but patients with wild-type *K-ras* did.^13,14^ For patients with mutated *K-ras* tumors, cetuximab and supportive care alone had numerically similar median OS (4.5 months and 4.6 months). For patients with wild-type *K-ras* tumors, cetuximab was associated with a > 4-month absolute improvement in median OS, compared with supportive care alone (9.5 months, *v* 4.8 months).^13^ In stratified analyses, quality-of-life benefit of cetuximab was restricted to the wild-type *K-ras* group.^14^

### Measure of Time Toxicity

As previously described, we considered any day with physical health care system contact as a time toxic day.^3^ In this pragmatic approach, a 1-hour visit for bloodwork, a 3-hour visit for chemotherapy, a 6-hour urgent care visit for dehydration, an emergency department visit, a day admitted in the hospital, or a day in a rehabilitation facility are all coded as a time toxic day. If a patient had multiple physical interactions with the health care system—bloodwork, clinic visit, chemotherapy infusion, and an infusion reaction resulting in an emergency department visit and subsequent discharge—on the same day, it was counted as a single time toxic day.

We calculated patient-level time toxicity (days with physical health care system contact) by analyzing treatment and follow-up forms and resource utilization assessment forms. The treatment and follow-up forms listed the dates of protocol-related health care contact (eg, bloodwork, infusions, imaging studies, etc). The resource utilization assessment form was completed every 28 days for each participant by trial staff, with the aim of collecting data on the number and type of medical resources consumed by each patient. The forms collected non–protocol-related office/clinic visits, outpatient procedures/treatments (such as imaging, transfusions, paracentesis, radiation, and chemotherapy), emergency department visits, hospitalizations, and admission to a facility (rehabilitation, long-term care, hospice, and others), along with the dates of each contact. If the exact date of an outpatient encounter was not recorded, we considered it to be on a separate day. The date(s) of an inpatient encounter (in hospital) were always available. For a time toxic day, we further classified the source of time toxicity as (1) planned and (2) unplanned, depending on if it was related to a per-protocol visit or an additional visit. If a day had both planned and unplanned time toxicities (eg, planned cetuximab leading to an infusion reaction leading to an emergency department visit), we treated it like planned time toxicity since it occurred first and was independent of future events.

A day without physical health care contact was considered a home day. Thus, a day could either be a time toxic day or a home day. For an individual patient, OS was the sum of time toxic days and home days. The resource utilization assessment forms also collected visits by clinicians to patients' homes and procedures (eg, scans and thoracenteses) performed when a patient was inpatient. We excluded these visits from time toxicity analyses since they either did not require patient travel to a health care facility or the inpatient day was already included as a time toxic day.

### Statistical Analysis

We compared medians of time measures (time toxic days, home days, and proportion of home days alive) across arms by a Wilcoxon test and stratified results by *K-ras* mutation status. All randomly assigned patients were included in the analyses on the basis of intention to treat. All reported *P* values are two-sided and were not adjusted for multiple testing. Participating centers received approval from their institutional ethics review boards. All patients provided written informed consent before participation.

## RESULTS

All trial participants (n = 572) had data on time toxicity available and were included in the current analyses. The median age was 63 years, 64% of participants were men, and 77% had an Eastern Cooperative Oncology Group performance status of 0-1. Baseline characteristics were balanced between arms, and detailed data are available in prior publications.^12-14^ Tumor *K-ras* mutation status was known for 394 patients (69%), 164 of whom (42%) tumors had mutated *K-ras*.

For the overall study population, median time toxic days were higher in the cetuximab arm (28, *v* 10, *P* < .001) and median home days were not statistically different between arms (140, *v* 121, *P* = .09; Table 1). The proportion of time toxic days (time toxic days/OS) was significantly more in the cetuximab arm (median 18%, *v* 6%, *P* < .001). For patients receiving cetuximab, of the 28 time toxic days, 14 (50%) were planned, that is, spent in receipt of systemic therapy and planned study procedures. Median time toxic days related to unplanned resource utilization were also higher in the cetuximab arm (8, *v* 5, *P* = .002). Appendix Table A1 (online only) presents details on the sources of unplanned time toxicity. Although median days hospitalized was 0 in both arms (Table 1), 42.2% of participants were ever hospitalized in the cetuximab arm (*v* 34.0% in the supportive care alone arm), with a median length of stay of 10 days.

**TABLE 1.**
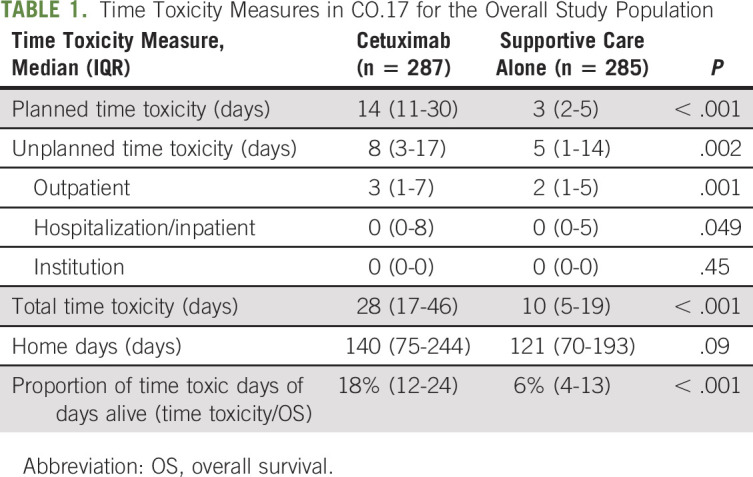
Time Toxicity Measures in CO.17 for the Overall Study Population

Results stratified by *K-ras* status are presented in Table 2. For patients with *K-ras*–mutated tumors, median home days were almost numerically equal in both arms (114 days *v* 112 days), with the cetuximab arm experiencing higher time toxicity (median 23 days, *v* 11 days, *P* < .001). For patients receiving cetuximab, of the 23 time toxic days, 11 (approximately 50%) were planned. Almost all numeric survival benefits associated with cetuximab (absolute 14 days difference) were essentially time toxic (absolute 12 days difference).

**TABLE 2.**
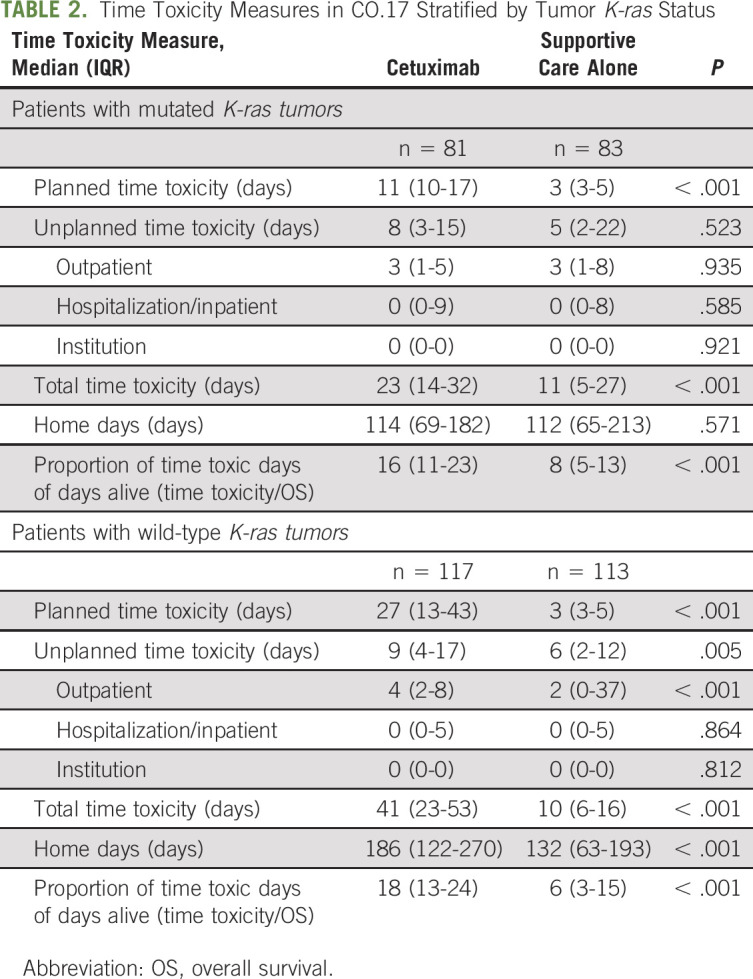
Time Toxicity Measures in CO.17 Stratified by Tumor *K-ras* Status

For patients with *K-ras* wild-type tumors, median home days were significantly higher in the cetuximab arm (186, *v* 132, *P* < .001), and so were time toxic days (41, *v* 10, *P* < .001). For patients in the cetuximab arm, 66% (27 of 41) of time toxic days were planned.

## DISCUSSION

In this secondary analysis of the CO.17 trial, we demonstrate that a measure of patient-level time toxicity can be successfully extracted. In CO.17, despite a statistically significant OS benefit with cetuximab in the overall study population, the number of home days was similar across arms and patients receiving cetuximab spent three times as many days alive with health care contact compared with patients treated with supportive care alone. In the *K-ras* wild-type group, which we now know derives the greatest benefit from cetuximab, cetuximab led to a significant increase in home days. Measures of time toxicity—especially in the setting of advanced cancer—may be helpful to guide patient-oncologist therapeutic decision making.

The primary objective of this work was to demonstrate the feasibility of collecting and comparing time toxicity from a completed RCT. We have previously calculated the estimated time toxicity of treatments from trial protocols and publications, but this work was limited by the lack of patient-level data.^3^ There are a few reasons we selected the CO.17 trial. First, the control arm was a supportive care–alone arm. With minimal protocol-mandated visits, time toxicity in this arm is a good surrogate of the baseline cancer-associated time toxicity. Second, the primary end point of the CO.17 trial was OS, and the median survival benefit was modest. This offers an opportunity to explore how the time toxicity of a treatment could erode into its survival benefit. Third, to our knowledge, CO.17 was the first CCTG trial to collect resource utilization data (eg, dates of health care contact) for a planned health economic analysis. Cooperative group trials that have incorporated measures of resource utilization are well-positioned to undertake such secondary analyses although these represent a minority of all trials. Even when trials collect resource utilization, post hoc time toxicity analysis may not be possible because of data structure and quality, and the resources and investments required. Ideally, time toxicity should be prospectively collected in future clinical trials. We are cognizant of the additional burdens that this can impose on the clinical trial team.^11^ Innovative primarily passive approaches can capture the required elements through (1) linking claims and trial data, even for a subpopulation of the trial (eg, Canadian provincial data and Medicare data) and (2) using mobile health technology (eg, geolocation services and sensor data) to track visits to a health care facility. Claims data have high accuracy and can provide longer follow-up than even primary trial data.^15^ We purposefully designed the current time toxicity metric—days with physical health care contact—to be objective and categorical. It is dichotomous and does not require adjudication. Some previous work on time burdens of cancer care has attempted to capture the exact time spent in travel, in waiting rooms, in infusions, etc.^7^ Although we broadly support such research, recording and reporting these in trials will be extremely burdensome.

The primary finding of this work is that despite a statistically significant OS benefit with cetuximab, time toxicity eroded into its survival benefit. In the overall population, home days were similar across arms. Results were even more striking for patients with *K-ras*–mutated tumors, where any numeric survival benefit associated with cetuximab was time toxic. One can argue that improving survival is not the only reason patients undergo treatment; some treatments are initiated to palliate symptoms (improve quality of life). However, even in this scenario, one expects truly effective treatments to be associated with less time toxicity by decreasing health care utilization. Patients value different things, and even after informed discussions, patients may elect to proceed with time toxic treatments, which is perfectly acceptable. We believe that clinicians should have the information to adequately inform and counsel patients about treatment decisions. Decision science should evaluate how best to present time toxicity data alongside traditional survival data. It is intuitive that the following two statements might affect patient perceptions of and ultimate decisions regarding treatment:

- Patients receiving this treatment on average live 6 weeks longer.
- Patients receiving this treatment on average live 6 weeks longer. However, patients getting the treatment spend more time coming to clinic and in the hospital—almost one in every 5th day will be spent in the clinic or hospital. Overall, patients getting the treatment spend the same number of days at home as patients focusing on symptom control alone.

For truly effective treatments, time toxicity data can support treatments. At the time that CO.17 was conducted (2003-2005) and when initial survival results were first published (2007), the impact of *K-ras* status on cetuximab benefit was unknown. Now, cetuximab is only clinically indicated and used in people with *K-ras* (and extended *RAS*) wild-type tumors. In CO.17, for people with *K-ras* wild-type tumors, cetuximab offered a substantial benefit in median home days (186, *v* 132); this may support a patient's decision to pursue treatment.^13^ Supplementing this point, quality-of-life data from CO.17 also demonstrated that benefits were restricted to people with *K-ras* wild-type tumors.^14^

In addition to the overall time toxicity data, the source of time toxicity can provide valuable insights. We found that planned visits accounted for approximately 50% of all time toxicity (and two thirds of the time toxicity for people with *K-ras* wild-type tumors) in the cetuximab arm. Cetuximab was administered weekly in CO.17. Despite encouraging data supporting the use of cetuximab administration every 2 weeks as far back as 2008,^16,17^ this every 2‐week schedule of administration only received US regulatory approval in 2021.^18^ Compared with weekly infusions, every 2‐week infusions would decrease at least two time toxic days a month and save precious resources and patient time.^19^ Clinical trials can be more onerous with additional visits for monitoring and research purposes; the 50% rate of planned time toxicity may represent the burdens that clinical trials impose on participants over what an individual might experience in standard-of-care clinical care. It is possible that measuring time toxicity in trials may falsely inflate true time burdens in care. Thus, clinical trial and real-world data can be complementary in understanding eventual time burdens faced by patients. Providing the minimum expected time toxicity over a treatment cycle/course (ie, the planned time toxicity of clinical trial or standard-of-care treatment options) to patients can aid with decision making. In addition, median unplanned time toxicity was numerically only a little higher in the cetuximab arm (8, *v* 5 days), mirroring the experience of the trial team that most cetuximab-related adverse events (eg, infusion reactions and rashes) could often be handled at planned visits. The background rate of unplanned time toxicity reflects health care utilization from the underlying cancer, which was only approximately 5% of days alive in the supportive care arm. Finally, details on the distribution of outpatient time toxicity (eg, lab visit, scan, infusion etc) can (1) provide more clinical nuance and (2) guide local improvement efforts to address time toxicity—such as home-based care when safe, feasible, and acceptable and care coordination to consolidate appointments.

This work has several limitations. First, the current measure itself is imperfect. It does not capture time toxicity when the patient is home (eg, telemedicine and home-based infusions), associated with care logistics (eg, time spent on the phone with insurance company), and for care partners. It does not differentiate between the source and quality of contact days. It assumes that all home days are equally good, and all time toxic days are equally bad. A more nuanced measure, perhaps a quality-weighted time toxic days, is appealing, but we must consider the complexity in collecting, calculating, collating, and communicating it. Measures such as Q-TWiST (quality-adjusted time without symptoms of disease or toxicity) face an uphill battle for routine adoption because of these reasons.^20^ The current measure is specifically optimized from a practical perspective and requires prospective validation. It does not make assumptions about how individual patients may interpret the time toxicity data, but provides objective data for patients to apply to their own values. Second, for some outpatient visits, the exact date of the encounter was missing. We considered these visits to be on a separate day. Thus, the time toxicity data in this analysis may represent a worst-case scenario. However, this assumption was common across arms and should not affect comparisons. In addition, the resource utilization assessment form was completed every 28 days for each patient, appropriately capping possible time toxic days (28 for each form) and preventing gross overestimation. Third, we did not include home visits by clinicians as time toxic since patients were not required to travel to a health care facility for such visits. However, even if included, these visits would be unlikely to affect overall results since more than 90% of forms noted zero home visits (detailed data not presented). Finally, well-intentioned efforts on decreasing time toxicity could have unintended consequences of worsening disparities in care access for vulnerable persons, who already face structural barriers to care.

In conclusion, we demonstrate that measures of time toxicity can be successfully extracted through secondary analyses of completed RCTs. Information on time toxicity can supplement traditional survival end point reporting in clinical trials. Future work should refine the time toxicity measure and explore how to deploy it prospectively in clinical trials.

## Figures and Tables

**TABLE A1.**
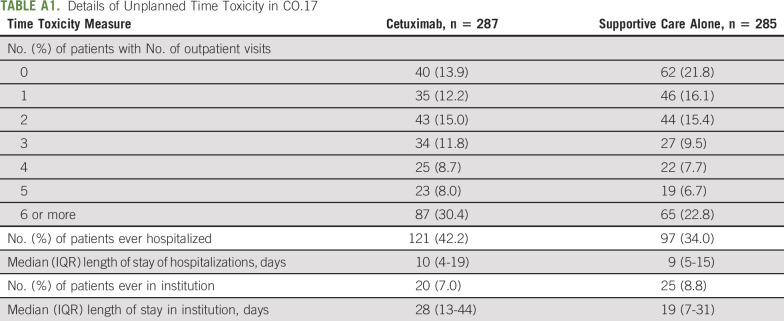
Details of Unplanned Time Toxicity in CO.17

## References

[1] Shen C Tannenbaum D Horn R : Overall survival in phase 3 clinical trials and the surveillance, epidemiology, and end results database in patients with metastatic colorectal cancer, 1986-2016: A systematic review. JAMA Netw Open 5:e2213588, 2022 35608860 10.1001/jamanetworkopen.2022.13588 PMC9131746

[2] Bange EM Doucette A Gabriel PE : Opportunity costs of receiving palliative chemotherapy for metastatic pancreatic ductal adenocarcinoma. JCO Oncol Pract 16:e678-e687, 2020 32130074 10.1200/JOP.19.00328 PMC7427417

[3] Gupta A Eisenhauer EA Booth CM: The time toxicity of cancer treatment. J Clin Oncol 40:1611-1615, 2022 35235366 10.1200/JCO.21.02810

[4] Gupta A Jensen EH Virnig BA Beg MS: Time-related burdens of cancer care. JCO Oncol Pract 18:245-246, 2022 34709950 10.1200/OP.21.00662

[5] Fundytus A Prasad V Booth CM: Has the current oncology value paradigm forgotten patients' time? Too little of a good thing. JAMA Oncol 7:1757-1758, 2021 34436535 10.1001/jamaoncol.2021.3600

[6] Sedhom R Samaan A Gupta A: Caregiver burden #419. J Palliat Med 24:1246-1247, 2021 34339334 10.1089/jpm.2021.0244

[7] Lim SA Hao SB Boyd BA : Opportunity costs of surgical resection and perioperative chemotherapy for locoregional pancreatic adenocarcinoma. JCO Oncol Pract 18:302-309, 2022 34709961 10.1200/OP.21.00311

[8] Rocque GB Williams CP Ingram SA : Health care-related time costs in patients with metastatic breast cancer. Cancer Med 9:8423-8431, 2020. 32955793 10.1002/cam4.3461 PMC7666754

[9] Lamarca A Palmer DH Wasan HS : Second-line FOLFOX chemotherapy versus active symptom control for advanced biliary tract cancer (ABC-06): A phase 3, open-label, randomised, controlled trial. Lancet Oncol 22:690-701, 2021 33798493 10.1016/S1470-2045(21)00027-9 PMC8082275

[10] Stupp R Mason WP van den Bent MJ : Radiotherapy plus concomitant and adjuvant temozolomide for glioblastoma. N Engl J Med 352:987-996, 2005 15758009 10.1056/NEJMoa043330

[11] Bennett CL Golub R Waters TM : Economic analyses of phase III cooperative cancer group clinical trials: Are they feasible? Cancer Invest 15:227-236, 1997 9171857 10.3109/07357909709039720

[12] Jonker DJ O'Callaghan CJ Karapetis CS : Cetuximab for the treatment of colorectal cancer. N Engl J Med 357:2040-2048, 2007 18003960 10.1056/NEJMoa071834

[13] Karapetis CS Khambata-Ford S Jonker DJ : K-ras mutations and benefit from cetuximab in advanced colorectal cancer. N Engl J Med 359:1757-1765, 2008 18946061 10.1056/NEJMoa0804385

[14] Au HJ Karapetis CS O'Callaghan CJ : Health-related quality of life in patients with advanced colorectal cancer treated with cetuximab: Overall and KRAS-specific results of the NCIC CTG and AGITG CO.17 trial. J Clin Oncol 27:1822-1828, 2009 19273701 10.1200/JCO.2008.19.6048

[15] Hanna TP Nguyen P Pater J : Can administrative data improve the performance of cancer clinical trial economic analyses? JCO Oncol Pract 15:e807-e824, 2019 10.1200/JOP.18.00691 31306036

[16] Ramanathan RK: Alternative dosing schedules for cetuximab: A role for biweekly administration? Clin Colorectal Cancer 7:364-368, 2008 19036688 10.3816/CCC.2008.n.048

[17] Tabernero J Pfeiffer P Cervantes A: Administration of cetuximab every 2 weeks in the treatment of metastatic colorectal cancer: An effective, more convenient alternative to weekly administration? Oncologist 13:113-119, 2008 18305055 10.1634/theoncologist.2007-0201

[18] Parikh AR Gonzalez-Gugel E Smolyakova N : Efficacy and safety of cetuximab dosing (biweekly vs weekly) in patients with KRAS wild-type metastatic colorectal cancer: A Meta-analysis. Oncologist 27:371-379, 2022 35522557 10.1093/oncolo/oyab030 PMC9074967

[19] Kasi PM: Call for adoption of synchronized biweekly dosing of anti-EGFR agent cetuximab: Implications for patients with metastatic colorectal cancer, and squamous cell carcinoma of the head and neck. Oncologist 27:336-337, 2022 35403691 10.1093/oncolo/oyac070 PMC9074985

[20] Gelber RD Cole BF Gelber S : Comparing treatments using quality-adjusted survival: The Q-Twist method. Am Statistician 49:161-169, 1995

